# The value of multi-sequence magnetic resonance imaging and whole-tumor apparent diffusion coefficient histogram analysis in differentiating p53 abnormal from non-p53 abnormal endometrial carcinoma

**DOI:** 10.3389/fonc.2025.1565152

**Published:** 2025-04-15

**Authors:** Yuying Sun, Jieying Zhang, Yilin Wang, Xinxin Zhang, Yan Chen

**Affiliations:** Radiology Department, National Cancer Center/National Clinical Research Center for Cancer/Cancer Hospital, Chinese Academy of Medical Sciences and Peking Union Medical College, Beijing, China

**Keywords:** endometrial cancer, magnetic resonance imaging, p53, diffusion-weighted imaging, histogram analysis

## Abstract

**Objective:**

To investigate the utility of multi-sequence magnetic resonance imaging (MRI) and whole-tumor apparent diffusion coefficient (ADC) histogram metrics in preoperatively differentiating p53 abnormal (p53abn) from non-p53abn endometrial carcinoma (EC).

**Methods:**

This retrospective study included 146 EC patients (29 p53abn cases and 117 non-p53abn cases) who underwent preoperative MRI scans. MRI features were analyzed. Whole-tumor ADC histogram analysis was conducted by delineating regions of interest (ROIs) on diffusion-weighted imaging (DWI) scans. Receiver operating characteristic (ROC) curve analysis with the area under the curve (AUC) was used for diagnostic performance evaluation.

**Results:**

Extrauterine extension (p=0.004) and lymphadenopathy (p=0.005) were more frequently observed in p53abn EC compared to non-p53abn EC. p53abn EC exhibited significantly lower value of minADC (p=0.001), meanADC (p=0.005), P10 (p=0.009), P50 (p=0.007), and P90 (p=0.013) ADC and higher value of kurtosis (p=0.008), compared to non-p53abn EC. MinADC demonstrated the highest discrimination ability in differentiating p53abn from non-p53abn EC [AUC 0.70(0.60;0.80)].

**Conclusion:**

Preoperative multi-sequence MRI findings and whole-tumor ADC histogram metrics are conducive to differentiating p53abn from non-p53abn EC.

## 1 Introduction

Endometrial cancer (EC) represents the most prevalent gynecological malignancy in developed nations, with a globally increasing incidence and a notable trend towards younger patient populations (1). In recent years, substantial progress has been achieved in the management of EC, with molecular classification emerging as one of the most innovative advancements in this field. Numerous studies demonstrated that the molecular subtypes could independently prognosticate clinical outcomes while exhibiting significant prognostic value, particularly within high-risk cohorts of EC (2, 3). The World Health Organization (WHO) has established four distinct molecular subtypes of EC (4). Among them, patients with p53 abnormal (p53abn) EC exhibit a significantly higher incidence of lymph node metastasis and poorer prognosis. Additionally, emerging evidence suggests that patients with high-risk p53abn EC may derive benefit from the combination of chemotherapy and adjuvant external beam radiotherapy in the adjuvant treatment setting (3, 5–7). Therefore, distinguishing EC based on distinct molecular subtypes is of critical importance.

Magnetic resonance imaging (MRI) is the preferred modality for preoperative assessment of endometrial cancer (EC), playing a crucial role in guiding subsequent treatment strategies, surgical planning, and evaluating therapeutic outcomes. Additionally, diffusion-weighted imaging (DWI) and its quantitative apparent diffusion coefficient (ADC) provide significant insights into the assessment of myometrial invasion (8, 9), tumor histological subtypes and grading (8, 10–14), as well as lymph node metastasis of EC (9, 15). As highlighted by Restaino et al. (16), recent management guidelines for EC have begun to incorporate imaging strategies, particularly emphasizing the importance of MRI in conjunction with molecular classification.

While the preoperative assessment capabilities of MRI for EC are well recognized, the full potential of integrating MRI with molecular classification remain inadequately explored. Therefore, this study aims to investigate the application of multi-sequence MRI and the whole tumor ADC histogram indices for preoperative differentiation of p53abn from non-p53abn EC.

## 2 Materials and methods

### 2.1 Study population

**Figure 1:**
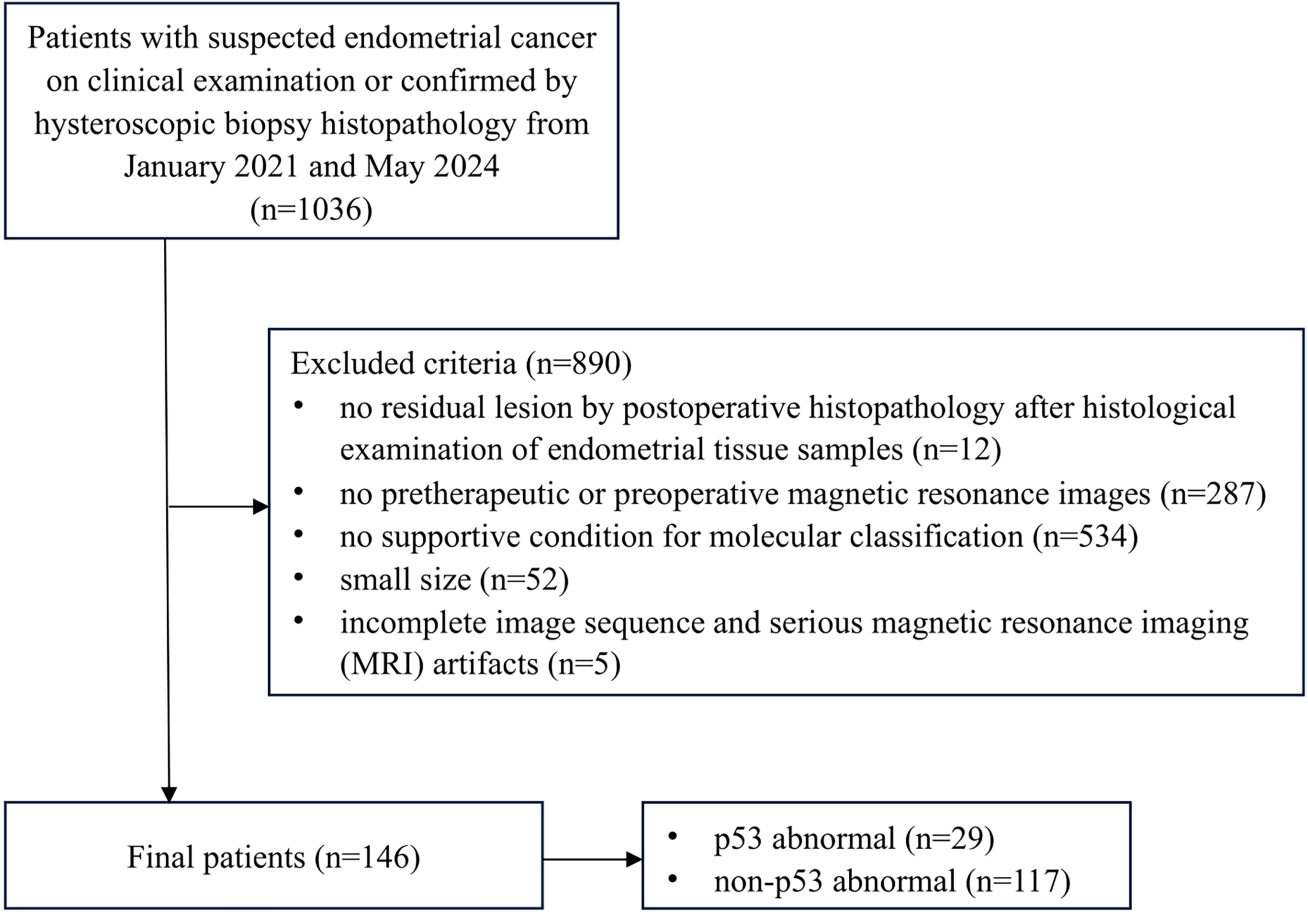
Flowchart illustrates the inclusion of study participants.

The research was approved by the relevant Institutional Review Board, and informed consent requirements were waived due to the retrospective nature of the study design. A total of 146 patients with EC confirmed by postoperative histopathology and preoperative MRI at our hospital were identified between January 2021 and May 2024. The inclusion criteria were as follows: (a) pelvic MRI examination performed before the operation, (b) standard surgery undergone at our hospital and no other neoadjuvant treatment received before the examination and the surgery, and (c) complete postoperative pathologic results. Excluded criteria were as follows: (a) no residual lesion according to the pathological result after histological examination of endometrial tissue samples (n=12), (b) unavailable pretherapeutic or preoperative MR images (n=287), (c) incomplete immunohistochemical or genetic testing results precluded molecular classification(n=534), (d) the small size (maximum diameter <1cm) was detrimental to delineation of the region of interest (ROI) (n=52), and (e) incomplete image sequences or poor image quality that affected lesion observation and data measurement (n=5). **Figure 1** illustrates the study flowchart.

### 2.2 MRI protocol

All patients underwent pelvic dynamic contrast enhanced (DCE) MRI on five 3.0-T MR scanners (three from GE Healthcare and two from Siemens Healthcare), using 8 channel-phased array body coils. The imaging protocol included five non-enhanced sequence: axial T1-weighted fast/turbo spin echo (T1-FSE/TSE), sagittal T2-weighted fast/turbo spin echo (T2-FSE/TSE), axial T2-FSE/TSE, axial T2-weighted fat-suppressed fast/turbo spin echo (T2-fs-FSE/TSE), and axial diffusion-weighted imaging (DWI). Additionally, three enhanced sequences were acquired in the axial, sagittal, and coronal planes. Detailed scanning parameters are provided in **Table 1**.

**Table 1:** MRI sequence parameters.

Parameters	Axial T1-FSE/TSE	Sagittal T2-FSE/TSE	Axial T2-FSE/TSE	Axial T2-fs-FSE/TSE	DWI	Sagittal LAVA/VIBE +C	Axial LAVA/VIBE +C	Coronal LAVA/VIBE +C
Repetition time (ms)	4~6	4529~6350	4700~6350	3160~6054	2291~4794	3~5	4~8	3~4
Echo time (ms)	1~3	85~120	87~120	81~92	58~76	1~2	2~4	1~2
Field of view (cm)	34~42	22~24	18~24	28~40	30~40	26~32	29~36	26~40
Matrix size	320×256	320×256	320×256	320×256	128×160	350×350	350×350	350×350
Slice thickness (mm)	4	4	4	5~6	4~6	1.5~4	1	1.5~4
Slice gap (mm)	1.0	0.4~1.0	1.0	1.0	0.3	0	0	0
b value (s/mm^2^)					0, 1000			

Enhanced scan was done by injecting gadopentetate dimeglumine into the upper limb vein by using a high-pressure syringe, with a flow rate at 2.0 ml/s and a total dose of 0.2 mmol/kg body weight. A total of 15 phases were obtained post-drug injection with a time interval of 15s in the sagittal plane, followed by a delayed phase with axial and coronal scanning.

MRI, magnetic resonance imaging; T1-FSE/TSE, T1-weighted fast/turbo spin echo; T2-FSE/TSE, T2-weighted fast/turbo spin echo; T2-fs-FSE/TSE, T2-weighted fat-suppressed fast/turbo spin echo; DWI, diffusion-weighted imaging.

### 2.3 Imaging processing and analyses

All MR images were independently reviewed by two radiologists (Reader 1 with 3 years of experience and Reader 2 with 30 years of experience in interpreting genitourinary MR images). The reviewers were aware that all patients had EC but were blinded to the clinical information and histopathological examination results.

The reviewers assessed multiple aspects of tumor presentation and characteristics, including size, depth of myometrial invasion, cervical stromal invasion, extrauterine extension, rectal or bladder invasion, peritoneal dissemination, presence of metastatic lymph nodes, and distant metastases. The maximum tumor diameter was measured on the T2-weighted imaging (T2WI). Deep myometrial invasion was defined as tumor involvement exceeding 50% of the myometrial thickness. On DCE-MRI, deep myometrial invasion was assessed during the equilibrium phase (2-3 minutes post-injection) because the tumor-to-myometrial contrast ratio was highest at this time point (17, 18). Cervical stromal invasion was evaluated in the delayed phase (4-5 minutes post-injection) (19). Lymphadenopathy was defined as a lymph node with a short-axis diameter greater than 8 mm (20).

**Figure 2:**
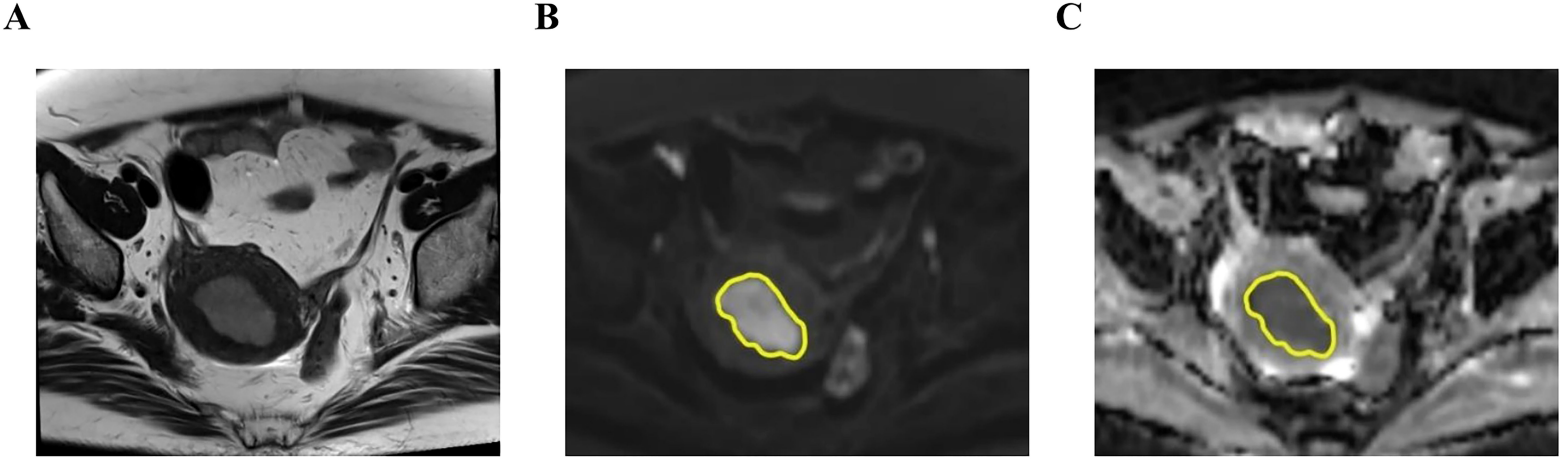
A 53-year-old woman with p53 abnormal endometrial cancer (endometrioid carcinoma, G3, stage IICm_p53abn_). The tumor shows slight hyperintensity on axial T2-weighted imaging (T2WI) **(A)**, hyperintensity on axial diffusion-weighted imaging (DWI) (b=1000s/mm^2^) **(B)**, and hypointensity on apparent diffusion coefficient (ADC) map **(C)**. The region of interest (ROI) was drawn along the contour of tumor on DWI and subsequently transferred to the corresponding ADC maps through an automated process (yellow in **B, C**).

**Figure 3:**
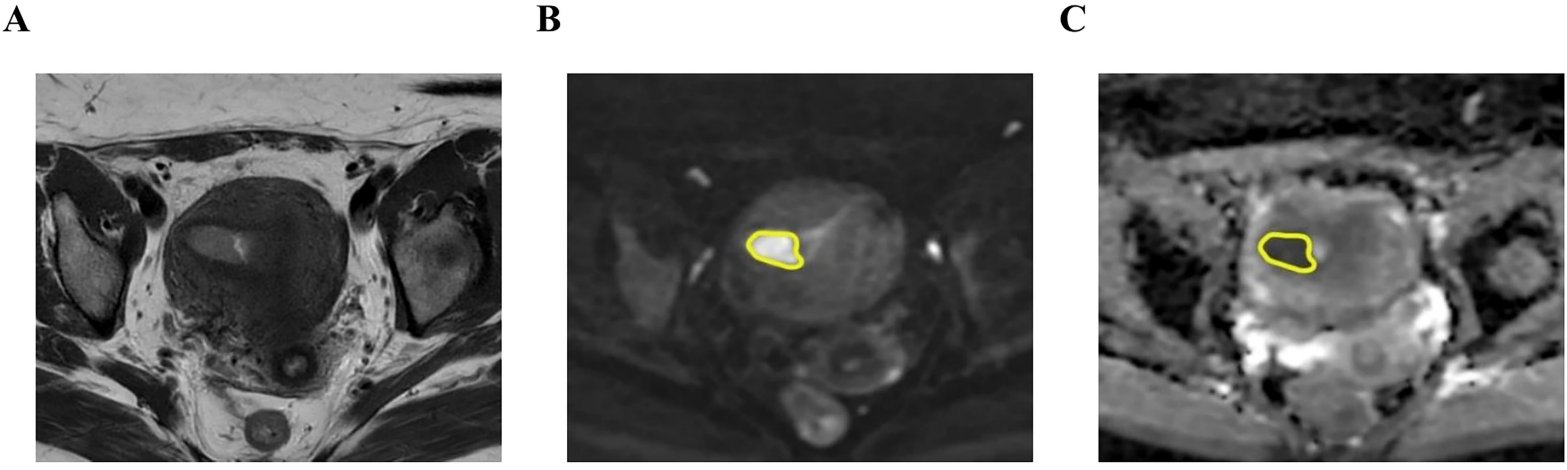
A 52-year-old woman with non-p53 abnormal endometrial cancer (endometrioid carcinoma, G2, stage IA2). The tumor shows slight hyperintensity on axial T2-weighted imaging (T2WI) **(A)**, hyperintensity on axial diffusion-weighted imaging (DWI) (b=1000s/mm^2^) **(B)**, and hypointensity on apparent diffusion coefficient (ADC) map **(C)**. The region of interest (ROI) was drawn along the contour of tumor on DWI and subsequently transferred to the corresponding ADC maps through an automated process (yellow in **B, C**).

The DWI images were transferred into Workstation (AW 4.6; GE Healthcare) for post-processing. ADC maps were generated from the DWI sequence using the Function tool ADC software. DWI with a b-value of 1000s/mm^2^ were used for image segmentation (**Figures 2**, **3**). Care was taken to exclude necrotic areas and vessels by referencing T2WI and DCE sequences.

For the single-section region of interest (ROI) of DWI analysis in the slice with the maximum diameter possible, the measurement of ADC value was performed by two radiologists independently on the workstation. The ROIs were drawn along the contour of tumor on DWI. The ROI was automatically transferred to the corresponding ADC maps and then mean ADC value of ROI was recorded. The ADC values measured by two radiologists were averaged for further statistical analysis.

For the whole-tumor volume of interest (VOI) analysis, MRI data were processed with 3D-slicer software (v.5.6.2; www.slicer.org; open-source software). One radiologist (Reader 1) delineated the tumor VOIs of all the patients. To assess interobserver variability, another radiologist (Reader 2), who was blinded to Reader 1’s results, independently delineated the VOIs for a randomly selected subset of 50 patients. VOI was drawn manually by tracing tumor boundaries on each slice containing tumor on the DWI. VOIs were then copied and pasted onto the corresponding ADC maps, and ADC histogram analysis was performed using Radiomics in the extension of 3D-slicer. The following parameters were extracted from the ADC histograms (1): meanADC, minADC, and maxADC; (2) 10th, 50th, and 90th percentiles of ADC (denoted as P10, P50, and P90 ADC, respectively); and (3) skewness, uniformity, entropy, and kurtosis.

### 2.4 Histopathological analysis

All lesion specimens were obtained surgically and processed by experienced pathologists. The staging of EC was conducted according to the latest International Federation of Gynecology and Obstetrics (FIGO) 2023 staging criteria (21). Molecular classification was performed in accordance with the European Society for Medical Oncology (ESMO) guidelines (22). Histological subtype, grade, and lymphovascular space invasion (LVSI) were confirmed by hematoxylin-eosin staining. The expression of p53 and mismatch repair (MMR) proteins was determined by immunohistochemical staining. All patients underwent genetic testing for POLE hotspot mutations, while some also underwent testing for MMR-related genes and p53 gene mutations. In cases of conflicting results between genetic tests and immunohistochemistry, priority was given to the immunohistochemical findings.

### 2.5 Statistical analysis

Statistical analyses were performed with SPSS statistical software (IBM Corp. version 26.0. Armonk, NY) and MedCalc statistical software (version 22.0. Mariakerke, Belgium). In cases where different results of categorical data were obtained, the reviewers discussed and reached a conclusion by consensus. The results achieved by consensus were used for the statistical analyses, except for interobserver agreement. Continuous variables are presented as median and interquartile range. The interobserver agreement for two readers’ measurements was analyzed by calculating intraclass correlation coefficient (ICC) with 95% confidence interval and ICC value greater than 0.75 indicates good correlation. Associations between MRI parameters and molecular classification were assessed using appropriate statistical tests. Chi-squared and Fisher’s exact tests were used to compare the differences in categorical variables. The normality of continuous variables was assessed by the Shapiro-Wilk (S-W) test. If p-value was greater than 0.05, the data were considered normally distributed. According to the normality of data distribution, continuous variables were compared using the independent samples t test or the Mann-Whitney U test. A two-tailed p-value less than 0.05 was considered to be statistically significant. The receiver operating characteristic (ROC) curve was used to evaluate the predictive performance of metrics. DeLong’s method was used to compare the differences in areas under the curves (AUCs). The maximum Youden index (sensitivity +specificity-1) was used to determine the optimal cutoff value and the corresponding sensitivity and specificity.

## 3 Results

### 3.1 Patients’ demographics

A total of 146 patients with EC were included in this study, who were divided into two groups with 29 p53abn and 117 non-p53abn. There was no statistically significant difference in age between the p53abn and non-p53abn groups (p=0.155). The FIGO stage of patients with non-p53abn EC was most frequently at stage I (n=72; 61.54%), while the FIGO stage IICm_p53abn_ was the most prevalent stage in patients with p53abn EC (n=18; 62.07%). In this study, 146 patients, including 122 patients with endometrioid adenocarcinoma, 6 with serous carcinoma, 2 with clear-cell carcinoma, 2 with mesonephric-like carcinoma, 9 with mixed carcinoma, and 5 with carcinosarcoma, were enrolled. More details are provided in **Table 2**.

**Table 2:** Clinical and pathologic characteristics of patients with endometrial cancer.

	Total	p53abn (n=29)	non-p53abn (n=117)				*p* value
			Total	POLEmut (n=14)	dMMR (n=37)	NSMP (n=66)	
Median age (years)	55.50 (50.00;62.00)	54.00 (49.00;58.50)	57.00 (51.00;62.00)	56.00 (50.00;59.25)	55.00 (51.00;64.50)	57.50 (50.75;62.00)	0.155
FIGO stage (2023)							0.010
I	72 (49.32)	0 (0)	72 (61.54)	13 (92.86)	16 (43.24)	43 (65.15)	
IA	60	0	60	13	12	35	
IA1	2	0	2	0	0	2	
IA2	45	0	45	0	12	33	
IA3	0	0	0	0	0	0	
IAm_POLEmut_	13	0	13	13	0	0	
IB	11	0	11	0	4	7	
IC	1	0	1	0	0	1	
II	46 (31.51)	18 (62.07)	28 (23.93)	0 (0)	15 (40.54)	13 (19.70)	
IIA	3	0	3	0	0	3	
IIB	1	0	1	0	0	1	
IIC	24	0	24	0	15	9	
IICm_p53abn_	18	18	0	0	0	0	
III	19 (13.01)	5 (17.24)	14 (11.97)	1 (7.14)	6 (16.22)	7 (10.61)	
IIIA	3	0	3	0	0	0	
IIIA1	3	0	3	0	1	2	
IIIA2	0	0	0	0	0	0	
IIIB	2	1	1	0	0	0	
IIIB1	0	0	0	0	0	0	
IIIB2	2	1	1	0	0	1	
IIIC	14	4	10	0	0	0	
IIIC1	4	1	3	0	1	2	
IIIC2	10	3	7	1	4	2	
IV	9 (6.16)	6 (20.69)	3 (2.56)	0 (0)	0 (0)	3 (4.55)	
IVA	0	0	0	0	0	0	
IVB	7	5	2	0	0	2	
IVC	2	1	1	0	0	1	
Histological subtype							<0.001
Endometrioid adenocarcinoma	122 (83.56)	18 (62.07)	104 (88.89)	12 (85.71)	31 (83.78)	61 (92.42)	
Low grade	86	11	75	5	17	53	
High grade	36	7	29	7	14	8	
Serous carcinoma	6 (4.11)	5 (17.24)	1 (0.85)	1 (7.14)	0 (0)	0 (0)	
Clear-cell carcinoma	2 (1.37)	0 (0)	2 (1.71)	0 (0)	1 (2.70)	1 (1.52)	
Mesonephric-like carcinoma	2 (1.37)	0 (0)	2 (1.71)	0 (0)	0 (0)	2 (3.03)	
Mixed carcinoma	9 (6.16)	3 (10.34)	6 (5.13)	1 (7.14)	4 (10.81)	1 (1.52)	
Carcinosarcoma	5 (3.42)	3 (10.34)	2 (1.71)	0 (0)	1 (2.70)	1 (1.52)	

Values are numbers (percentages) for categorical variables and median (interquartile range) for continuous variables. *p* values are calculated using a Chi-square test or Fisher’s exact test for categorical variables and an independent samples t test for continuous variables following normal distribution.

FIGO, International Federation of Gynecology and Obstetrics; p53abn, p53 abnormal; POLEmut, POLE-ultramutated; dMMR, mismatch repair deficient; NSMP, no specific molecular profile.

### 3.2 Differences in MRI findings and ADC histogram metrics between p53abn and non-p53abn EC

Extrauterine extension (p=0.004) and lymphadenopathy (p=0.005) were more common in p53abn EC compared to non-p53abn EC. p53abn EC showed significantly lower value of minADC (p=0.001), meanADC (p=0.005), P10 (p=0.009), P50 (p=0.007), and P90 (p=0.013) ADC and higher value of kurtosis (p=0.008) compared to non-p53abn EC. While, no significant differences were found in other MRI metrics and ADC histogram parameters between p53abn and non-p53abn EC (**Table 3**). Among the 50 randomly selected lesions, ICCs for all ADC histogram parameters were excellent, ranging from 0.92 to 0.98. Detailed results are shown in **Table 4**.

**Table 3:** Comparison of MRI findings and ADC histogram metrics.

	p53abn (n=29)	non-p53abn (n=117)	*p* value
Maximum tumor diameter (cm)	3.90 (3.15;5.65)	3.40 (2.10;4.85)	0.086
Deep myometrial invasion			0.693
No	20 (68.97)	85 (72.65)	
Yes	9 (31.03)	32 (27.35)	
Cervical stromal involvement			0.356
No	24 (82.76)	104 (88.89)	
Yes	5 (17.24)	13 (11.11)	
Extrauterine extension			0.004
No	21 (72.41)	109 (93.16)	
Yes	8 (27.59)	8 (6.84)	
Rectal or bladder invasion			–
No	29 (100)	117 (100)	
Yes	0 (0)	0 (0)	
Peritoneal dissemination			0.050
No	25 (86.21)	113 (96.58)	
Yes	4 (13.79)	4 (3.42)	
Lymphadenopathy			0.005
No	20 (68.97)	106 (90.60)	
Yes	9 (31.03)	11 (9.40)	
Distant metastases			0.359
No	28 (96.55)	116 (99.15)	
Yes	1 (3.45)	1 (0.85)	
ADC value*	0.82 (0.76;0.91)	0.86 (0.77;0.94)	0.154
MinADC	0.50 (0.37;0.60)	0.61 (0.50;0.72)	0.001
MeanADC	0.77 (0.70;0.86)	0.86 (0.75;0.94)	0.005
MaxADC	1.05 (0.94;1.29)	1.19 (1.05;1.36)	0.095
P10	0.65 (0.59;0.75)	0.72 (0.64;0.82)	0.009
P50	0.77 (0.69;0.87)	0.85 (0.75;0.93)	0.007
P90	0.92 (0.81;1.01)	0.98 (0.89;1.11)	0.013
Skewness	0.13 (-0.19;0.82)	0.37 (-0.11;0.63)	0.801
Uniformity	0.02 (0.01;0.03)	0.02 (0.01;0.04)	0.268
Entropy	5.87 (5.48;6.52)	5.94 (5.02;6.32)	0.402
Kurtosis	3.68 (2.93;5.47)	3.07 (2.68;3.99)	0.008

Values are numbers (percentages) for categorical variables and median (interquartile range) for continuous variables. *p* values are calculated using a Chi-square test or Fisher’s exact test for categorical variables, and a Mann-Whitney U test for continuous variables following non-normal distribution. ADC value* is measured in the slice with the maximum diameter possible. ADC values are in units of × 10^−3^ mm^2^/s.

MRI, magnetic resonance imaging; ADC, apparent diffusion coefficient; p53abn, p53 abnormal.

**Table 4:** Interobserver intraclass correlation coefficient between two readers for ADC histogram metrics.

Variable	ICC (95%CI)	*p* value
P10	0.98 (0.97;0.99)	< 0.001
P50	0.95 (0.92;0.97)	< 0.001
P90	0.93 (0.85;0.97)	< 0.001
MeanADC	0.97 (0.96;0.98)	< 0.001
MinADC	0.96 (0.93;0.97)	< 0.001
MaxADC	0.98 (0.97;0.99)	< 0.001
Skewness	0.98 (0.95;0.99)	< 0.001
Uniformity	0.95 (0.90;0.98)	< 0.001
Entropy	0.94 (0.87;0.97)	< 0.001
Kurtosis	0.92 (0.82;0.96)	< 0.001

ADC, apparent diffusion coefficient.

### 3.3 ROC analysis for differentiating p53abn from non-p53abn EC

**Figure 4:**
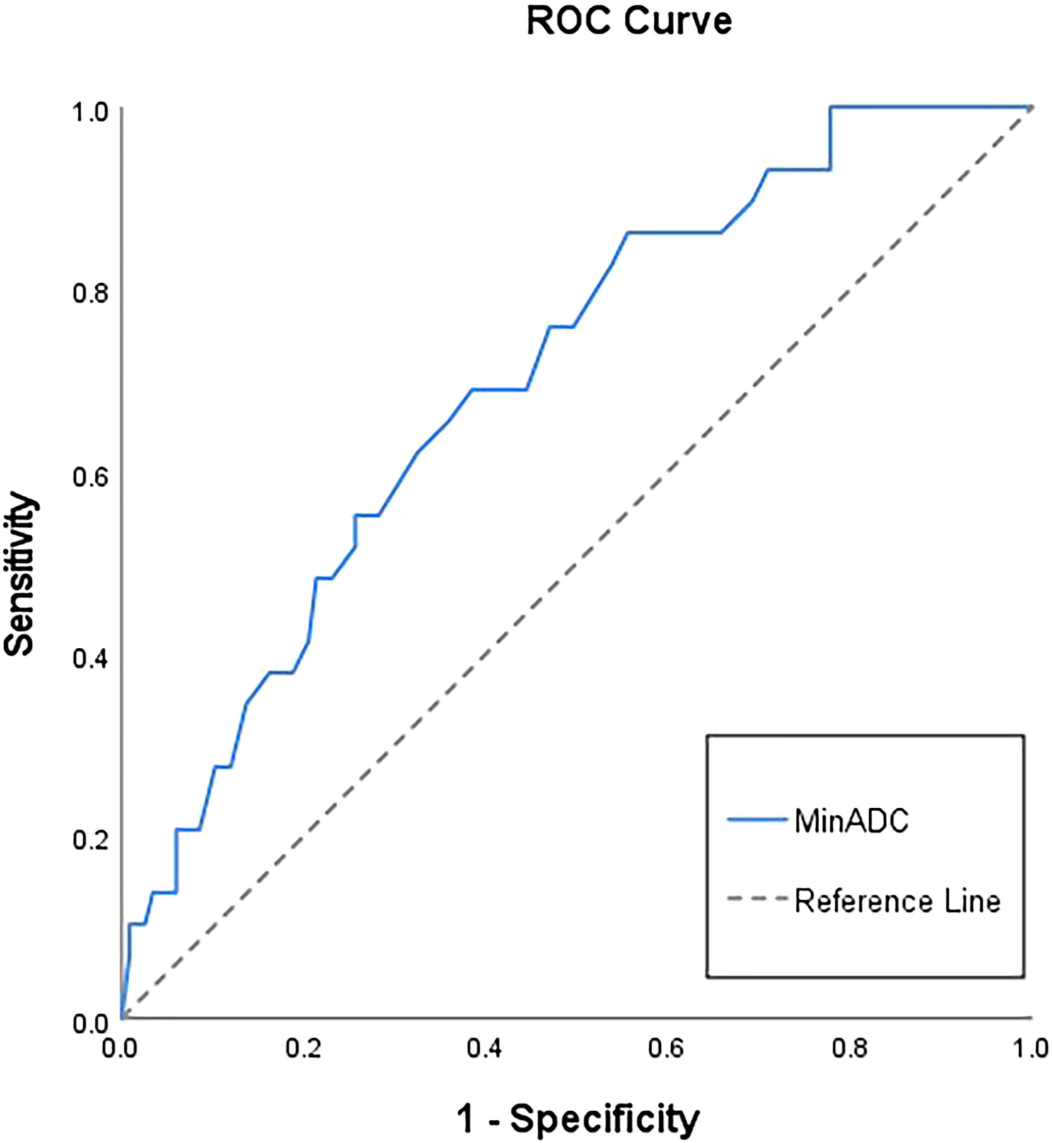
Receiver operating characteristic (ROC) curve of minADC to differentiate p53 abnormal from non-p53 abnormal endometrial cancer. The area under the curve (AUC) of minADC is 0.70(0.60;0.80).

In differentiating p53abn from non-p53abn EC, minADC achieved the largest AUC of 0.70 among the MRI findings and ADC histogram metrics, while the diagnostic efficiency of other indicators was mediocre in general (**Table 5**). However, there were no statistical differences in the AUC values among these indicators through the Delong test validation. The ROC curve of minADC is shown in **Figure 4**.

**Table 5:** The performance of MRI findings and ADC histogram metrics for differentiating p53abn from non-p53abn endometrial cancer.

	Youden index	Optimal cut-off value	Sensitivity (%)	Specificity (%)	AUC (95%CI)
Extrauterine extension	0.21	0.50	27.60	93.20	0.60 (0.48;0,73)
Lymphadenopathy	0.22	0.50	31.00	90.60	0.61 (0.48;0.73)
MinADC	0.31	0.63	86.20	44.40	0.70 (0.60;0.80)
MeanADC	0.31	0.85	75.90	55.60	0.67 (0.56;0.78)
P10	0.27	0.67	58.60	68.40	0.66 (0.55;0.76)
P50	0.26	0.78	58.60	67.50	0.66 (0.55;0.77)
P90	0.23	0.98	72.40	50.40	0.65 (0.54;0.76)
Kurtosis	0.28	2.78	89.70	38.50	0.66 (0.56;0.76)

MRI, magnetic resonance imaging; ADC, apparent diffusion coefficient; p53abn, p53 abnormal.

## 4 Discussion

Our study has identified distinct characteristics between p53abn and non-p53abn EC in terms of the frequency of invasive features and ADC histogram metrics. Extensive study of the surrogate markers on molecular classification of EC has shown a good relationship to clinical outcome, establishing their prognostic value. p53abn EC exhibit the poorest clinical outcome, irrespective of risk group, type of adjuvant treatment, tumor type or grade (23–25). Invasive features and advanced stage are frequently observed in p53abn EC, consistent with our study findings that extrauterine extension and lymphadenopathy were more prevalent in p53abn EC.

Molecular profiling traditionally depends on tissue obtained from surgical resection or biopsy in clinical practice. However, noninvasive imaging, through the identification of imaging markers correlating with molecular profiles, offers a promising frontier for tumor characterization. Although direct histopathological analysis remains the gold standard, preoperative prediction of molecular subtypes using MRI could have significant clinical implications. Relevant research reveals the potential of molecular classification of EC to improve patient management, such as reducing undertreatment in p53abn patients (25).

Our results demonstrated that quantitative ADC metrics, including minADC, meanADC, P10, P50, and P90 ADCs can effectively distinguish p53abn from non-p53abn EC. The ADC values of p53abn ECs were significantly lower than those of non-p53abn ECs, reflecting the more compact arrangement of tumor cells, smaller extracellular spaces, and restricted water molecule diffusion characteristic of p53abn EC. However, our study did not identify a statistically significant difference in ADC values between p53abn and non-p53abn EC. This may be attributed to tumor heterogeneity and the sampling bias inherent in single-slice analysis. Whole-tumor ADC histogram analysis facilitates the quantification of ADC value distribution, serving as a marker for structural heterogeneity and complexity. This approach mitigates sampling bias inherent in single-slice ROI delineation, thereby yielding more reproducible results compared to two-dimensional analyses (15, 16).

Our study demonstrated that kurtosis can effectively differentiate p53abn from non-p53abn EC. Kurtosis was significantly higher in p53abn EC compared to non-p53abn EC, likely due to increased tumor heterogeneity, which disperses cell characteristic values and elevates kurtosis. In addition, the application of new MRI imaging techniques, such as amide proton transfer weighting (APTw) imaging combined with intravoxel incoherent motion (IVIM), and radiomics are conducive to discriminating the p53abn status of EC (26, 27). These advancements may ultimately enhance our ability to noninvasively differentiate molecular subtypes, thereby facilitating a more refined approach to personalized treatment planning.

It is important to highlight that the application of these techniques extends beyond research on p53abn EC. The combination of APTw and IVIM has been demonstrated to be an effective non-invasive method for detecting microsatellite instability (MSI) in EC (28). Numerous studies have shown that radiomics analysis based on MRI or CT holds significant value in identifying MSI status in EC and predicting recurrence risk in patients with EC (29–32). In regions with limited access to MRI, CT radiomics models offer broader applicability. Furthermore, a recent study proposes the potential of using radiomics analysis of ultrasound images to predict molecular and genomic characteristics of endometrial cancer, aiming to provide a cost-effective and efficient approach for future diagnosis and treatment (33).

Our exploration of imaging characteristics based on molecular classification is preliminary and further research is still requisite for supplementation. Our study had some limitations. First, this was a retrospective, single-institution study with a limited sample size. A larger-scale study is needed to confirm these findings. Second, many patients underwent endometrial sampling biopsy (dilation and curettage, with or without hysteroscopy) prior to MRI, which may have reduced tumor size and potentially influenced its diagnostic performance. However, we excluded cases involving tumors that were either too small or not visible. Third, manual ROI segmentation introduces the potential for observer bias, highlighting the necessity for more advanced image analysis techniques, such as automatic or semiautomatic segmentation.

## 5 Conclusion

Preoperative multi-sequence MRI findings and whole-tumor ADC histogram analysis are helpful to differentiate p53abn from non-p53abn EC. This distinction is clinically valuable for accurate risk stratification and treatment planning. Identifying p53abn EC, which is often associated with more aggressive behavior, allows clinicians to tailor therapeutic strategies, thereby improving patient outcomes and optimizing the use of adjuvant therapies.

## Data Availability

The raw data supporting the conclusions of this article will be made available by the authors, without undue reservation.

## Glossary

ADC: apparent diffusion coefficient
APTw: amide proton transfer weighting
AUC: area under the curve
DCE: dynamic contrast enhanced
DWI: diffusion-weighted imaging
EC: endometrial cancer
ESMO: European Society for Medical Oncology
FIGO: International Federation of Gynecology and Obstetrics
ICC: intraclass correlation coefficient
IVIM: intravoxel incoherent motion
LVSI: lymphovascular space invasion
MMR: mismatch repair
MRI: magnetic resonance imaging
MSI: microsatellite instability
p53abn: p53 abnormal
POLEmut: POLE-ultramutated
ROC: receiver operating characteristic
ROI: region of interest
S-W: Shapiro-Wilk
T1-FSE/TSE: T1-weighted fast/turbo spin echo
T2-FSE/TSE: T2-weighted fast/turbo spin echo
T2-fs-FSE/TSE: T2-weighted fat-suppressed fast/turbo spin echo
T2WI: T2-weighted imaging
VOI: volume of interest
WHO: World Health Organization
