# Supplementary figures and images for: The value of multi-sequence magnetic resonance imaging and whole-tumor apparent diffusion coefficient histogram analysis in differentiating p53 abnormal from non-p53 abnormal endometrial carcinoma
Source: Front Oncol. 2025 Apr 15;15:1565152. doi: 10.3389/fonc.2025.1565152 (PMC12039310; doi:10.3389/fonc.2025.1565152)

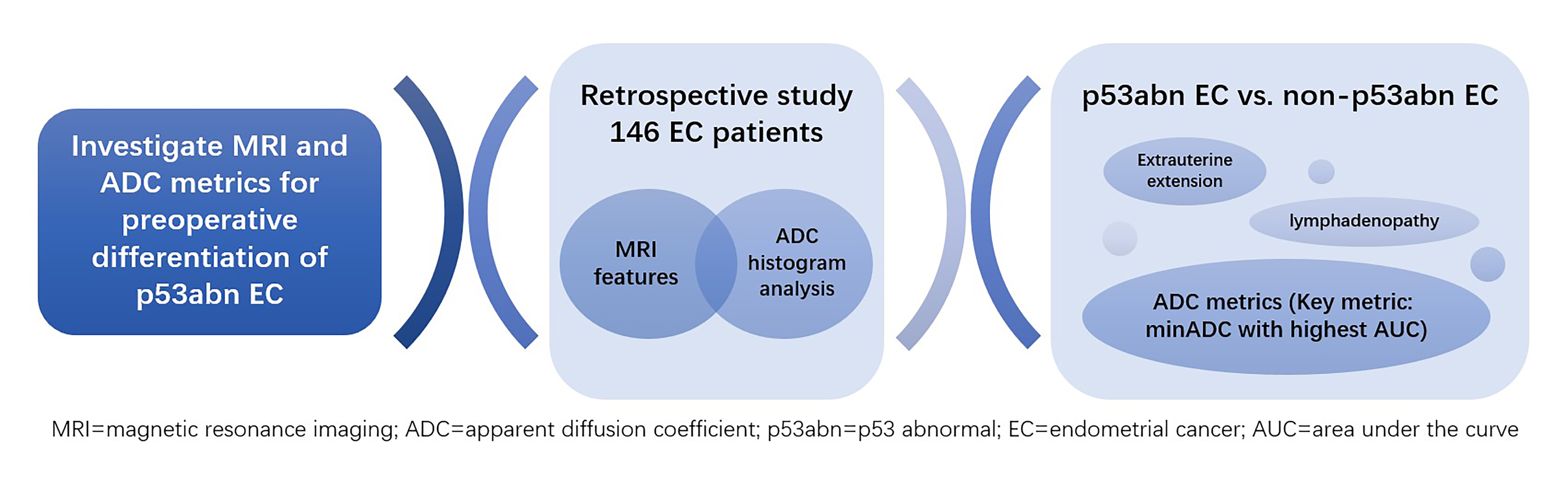

Supplement: Supplementary file 1 [file Image1.jpeg]
